# The Differential Diagnosis of Coma in the ICU: Hyperacute Postoperative Guillain‐Barré Syndrome—A Case Report

**DOI:** 10.1002/ccr3.72064

**Published:** 2026-02-26

**Authors:** Miron Tiganas, Abdulrahman Ismaiel, Dan Sebastian Dirzu, Iris Aszalos

**Affiliations:** ^1^ Department of Anesthesia and Intensive Care Cluj County Emergency Clinical Hospital Cluj‐Napoca Romania; ^2^ 2nd Department of Internal Medicine Iuliu Hatieganu University of Medicine and Pharmacy Cluj‐Napoca Romania; ^3^ STAR‐UBB Institute Babeș‐Bolyai University Cluj‐Napoca Romania

**Keywords:** cardiopulmonary arrest, delayed diagnosis, Guillain‐Barré syndrome, hypoxic encephalopathy, vascular surgery

## Abstract

Guillain‐Barré syndrome (GBS) is a rare, immune‐mediated neurological disorder that can be challenging to diagnose in postoperative patients due to atypical manifestations and overlapping conditions. This case report highlights the diagnostic and therapeutic challenges of GBS following vascular surgery. We present the case of a 56‐year‐old man scheduled for elective femoro‐popliteal bypass surgery. The initial neurological development of the patient's GBS was masked by delirium requiring sedation and culminated with cardio‐respiratory arrest in the 72 h following admission in the post‐operative care unit for surveillance. The patient subsequently developed a profound coma with flaccid quadriplegia. A search for the etiology of these symptoms was initiated, with a belated diagnosis of GBS, but with a positive, albeit partial, response to treatment by intravenous immunoglobulin administration. As in this case report, GBS in a post‐surgical setting should not be expected in its classical form, frequently characterized by a more severe clinical presentation regarding the onset of symptoms, associated dysautonomia and atypical, yet possible encephalopathy.

## Key Clinical Message

Guillain‐Barré syndrome should be considered in postoperative ICU patients presenting with coma, flaccid paralysis, or severe dysautonomia—even in the absence of typical infectious triggers—since hyperacute forms can mimic hypoxic brain injury and delay diagnosis.

## 1 Introduction

Guillain‐Barré syndrome (GBS) is a rare but serious neurological condition characterized by acute, immune‐mediated peripheral nerve damage [1]. It typically manifests as progressive motor weakness, areflexia, and, in severe cases, respiratory failure. While it is most commonly associated with preceding infections, GBS can also occur in a post‐surgical setting [2]. However, the postoperative presentation of GBS often deviates from the classical form, with an atypical onset and clinical features, making the diagnosis more challenging and frequently delayed [3].

In surgical and intensive care unit (ICU) settings, the diagnosis of GBS is further confounded by overlapping clinical conditions such as ICU‐acquired weakness, critical illness polyneuropathy, post‐anesthetic complications, and altered mental status due to delirium or sedatives. Additionally, postoperative GBS often presents with symptoms not traditionally associated with the syndrome, including dysautonomia, encephalopathy, and rapid neurological deterioration. These atypical manifestations demand heightened awareness among healthcare providers, as early recognition and intervention can significantly improve outcomes.

The unfolding clinical picture of GBS in postoperative patients is often complicated by confounding events that mislead the diagnostic process. In some cases, life‐threatening events such as cardio‐respiratory arrest may initially be attributed to alternative causes, as in this report where it was mistakenly assumed to have led to hypoxic encephalopathy. Profound coma, severe dysautonomia, and rapidly progressing quadriplegia, hallmarks of a severe GBS presentation, may emerge, but are easily overlooked or misinterpreted in this complex context. This underlines the importance of considering GBS in any postoperative patient with unexplained neurological or autonomic symptoms [2].

This case report highlights the diagnostic and therapeutic challenges associated with postoperative GBS following elective femoro‐popliteal bypass surgery. The patient's clinical course was marked by profound coma, flaccid quadriplegia, and severe dysautonomia, with delayed recognition of the underlying condition. This case underscores the need for vigilance and a multidisciplinary approach to diagnose and manage GBS in surgical patients presenting with atypical or severe symptoms.

## 2 Case Description

A 56‐year‐old male patient was admitted for elective right lower limb femoro‐popliteal bypass surgery, indicated for occlusive peripheral arterial disease secondary to an occluded right superficial femoral artery. His medical history was significant for multiple comorbidities, including severe ischemic heart disease, involving an acute myocardial infarction in 2007 necessitating percutaneous transluminal coronary angioplasty with stenting of the interventricular artery, and follow‐up evaluations in 2013 revealing 70%–80% occlusion post‐stenting, 70% occlusion of the diagonal artery, 85% occlusion of the right coronary artery, and 40% occlusion of the circumflex artery at its origin. Additionally, he had ischemic dilated cardiomyopathy, congestive heart failure classified as NYHA III with a mildly reduced ejection fraction, poorly controlled insulin‐dependent type 2 diabetes mellitus with neuropathy and peripheral artery disease as secondary complications, hypertension, grade III obesity, generalized anxiety disorder, and active tobacco use. Notably, there was no history of upper respiratory tract infections or gastroenteritis in the 4 weeks preceding hospitalization, and screening for influenza and COVID‐19 were negative upon admission. *It is to be mentioned that, although these comorbidities could have represented major contraindications for the surgery, a thorough discussion explaining the morbidity and mortality involved had been undergone with the patient, both by the surgeon and the anesthesiologist. The patient refused to accept the possibility of a limb amputation as a future treatment option and accepted the surgery, with all its complications*.

The procedure, performed under epidural anesthesia, was complicated by intraoperative thrombosis of the venous graft, necessitating reconstruction and extending the surgery duration to a total of 8 h.

### 2.1 Clinical Presentation

The immediate postoperative period was complicated by delirium, despite the implementation of both non‐pharmacological and pharmacological preventive measures from the day of surgery. Episodes of extreme agitation resulted in the displacement of the epidural catheter and surgical drain, necessitating continuous intravenous light sedation. Sedation was maintained for 48 h at the minimal dose required to achieve a Richmond Agitation‐Sedation Scale (RASS) score of 0, allowing the patient to be fed and mobilized into a sitting position. Mild muscular weakness observed during this period was attributed to pre‐existing diabetic and vascular peripheral neuropathies.

On the third postoperative day, the patient experienced apnea shortly after being assessed with a RASS score of −1, followed by cardiac arrest. Advanced cardiac life support (ACLS) was initiated according to the 2021 European Resuscitation Council guidelines, achieving return of spontaneous circulation (ROSC) after 10 min of asystole. Following stabilization, an extensive diagnostic workup was undertaken to determine the cause of the arrest. Electrocardiogram (ECG) traces and cardiac enzymes provided no evidence of malignant arrhythmias or myocardial infarction, and pulmonary embolism was excluded as the patient was under therapeutic anticoagulation for bypass graft patency. A computed tomography (CT) scan ruled out ischemic or hemorrhagic stroke but revealed findings consistent with probable aspiration pneumonia, leaving the cause of respiratory arrest unclear.

Twenty‐four hours after the cardiac arrest, with sedation discontinued, neurological evaluation using the FOUR scale yielded a score of 5, characterized by normal pupil and corneal reflexes. However, the patient exhibited flaccid quadriplegia with diminished deep tendon reflexes, without cranial nerve involvement. Seizure activity was absent, as confirmed by continuous eight‐channel electroencephalographic (EEG) monitoring initiated shortly after the arrest, which showed no epileptiform activity over the subsequent week. Despite profound coma, the patient remained on mechanical ventilation with stable oxygenation and adequate carbon dioxide elimination.

Hemodynamically, the patient demonstrated a hyperdynamic state, marked by persistent sinus tachycardia and arterial hypertension throughout his ICU stay. From the time of ICU admission, he presented with mild but persistent fever, which responded only modestly to antipyretic therapy. No evidence of additional organ dysfunction was identified during this period.

### 2.2 Diagnostic Assessment

**FIGURE 1:**
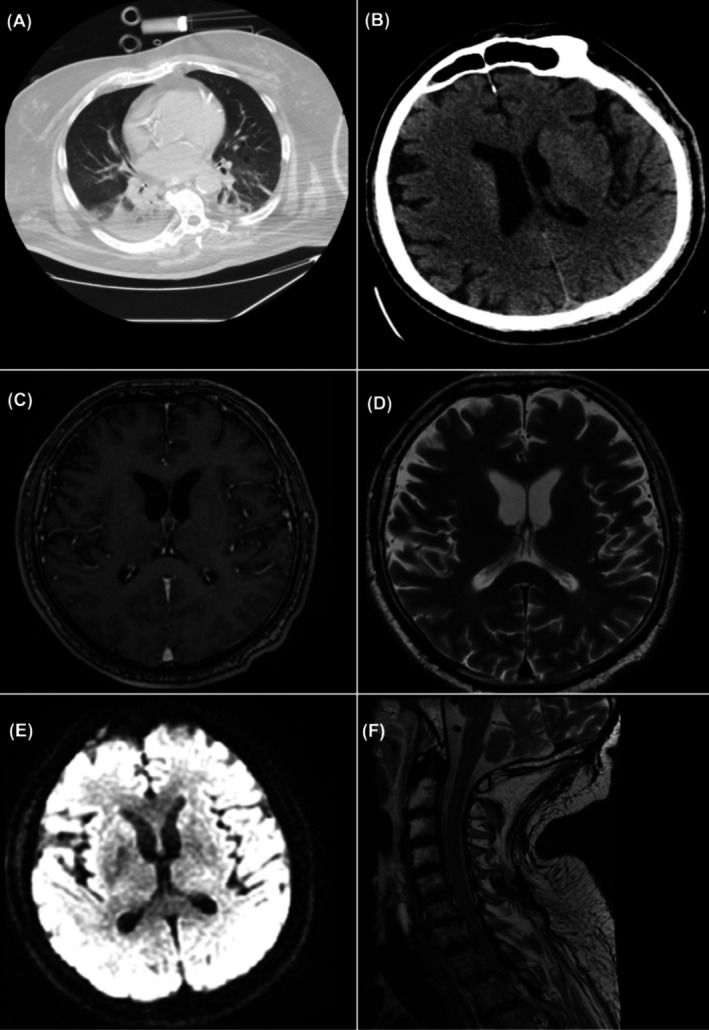
Cerebral and spinal magnetic resonance imaging. Note no pathological intracranial or spinal findings. (A) Chest CT (Axial, Lung Window): Patchy ground‐glass opacities predominantly in the lower lobes, more pronounced posteriorly, associated with bilateral atelectasis. Findings suggestive of aspiration pneumonia. (B) Non‐Contrast Head CT (Axial): Lateral ventricles displaying moderate cortical atrophy, but no signs of ischemia or space‐occupying lesions. (C) Brain MRI—T1‐weighted Post‐Contrast (Axial): No abnormal contrast enhancement. No signs of ring‐enhancing lesions or abnormal meningeal enhancement. No evidence of abscess, neoplasm, or active inflammation. (D) Brain MRI—T2‐weighted (Axial): No signs of encephalopathy or other significant pathological findings. (E) Brain MRI—Diffusion‐Weighted Imaging (DWI): No signs of myelopathy or other significant pathological findings. (F) Cervical Spine MRI—T2‐weighted Sagittal: Normal cervical alignment. No evidence of spinal cord compression, demyelination, or edema. The spinal cord shows normal signal intensity. No herniated discs or spinal canal stenosis visible in this slice. No MRI evidence of cervical myelopathy or demyelinating disease.

The neurology team recommended follow‐up with cerebral magnetic resonance imaging (MRI) to investigate potential hypoxic‐anoxic brain lesions, particularly in the midbrain or pons. However, due to technical difficulties associated with transporting and imaging the patient, the MRI was performed 72 h post‐cardiac arrest. The imaging studies revealed no pathological changes (Figure 1).

As outlined in Table 1, no significant findings indicative of metabolic coma were identified, aside from low‐grade fever, mild hypothyroidism, and moderate hypernatremia. Thiamine levels were normal. Neuroleptic malignant syndrome was ruled out, and administration of benzodiazepines did not elicit the Lazarus response, thereby excluding catatonia or akinetic mutism as potential causes, as advised by the internal medicine team.

**TABLE 1:** Blood test values done over the initial 48 h in the ICU, at the time of the respiratory and cardiac arrest.

Parameter	Patient value	Reference range
BIOCHEMISTRY		
**ALT**	**56 U/L**	**< 50 U/L**
**AST**	**73 U/L**	**< 50 U/L**
**CK**	**981 U/L**	**< 171 U/L**
CK‐MB	16 U/L	< 24 U/L
Creatinine	0.72 mg/dL	0.67–1.17 mg/dL
**CRP**	**34.29 mg/dL**	**< 0.5 mg/dL**
**Procalcitonin**	**0.165 ng/mL**	**< 0.06 ng/mL**
**Total Protein**	**6.15 g/dL**	**6.6–8.3 g/dL**
Urea	29 mg/dL	17–43 mg/dL
**Troponin I (high sensitivity)**	**626.1 pg/mL**	**< 19.8 pg/mL**
**Free—T4**	**0.66 ng/dL**	**0.92–1.68 ng/dL**
TSH	2.01 μU/mL	0.27–4.2 μU/mL
Thiamine	4 μg/mdL	2.5–7.5 μg/dL
Sodium	151 mmol/L	135–145 mmol/L
COAGULATION		
aPTT	35.3 s	23.5–36.5 s
**INR**	**1.33**	**0.79–1.16**
**Fibrinogen**	**959 mg/dL**	**200–400 mg/dL**
HEMATOLOGY		
**WBC**	**16.77 × 10^9/L**	**4–10 × 10^9/L**
**RBC**	**3.29 × 10^12/L**	**4.5–5.5 × 10^9/L**
**Hb**	**8.9 g/dL**	**13–17 g/dL**
**Hct**	**28.5%**	**40%–54%**
MCV	86.7 fL	80–95 fL
MCHbC	31.1 g/dL	31–36 g/dL
Platelets	305 **×** 10^9/L	150–400 **×** 10^9/L
**Neutrophils %**	**84.3%**	**30%–75%**
**Lymphocites %**	**8.7%**	**20%–45%**
IMMUNOLOGY		
GBS anti‐ganglioside antibodies		
Anti‐GM1 IgG/IgM	Negative	Negative
Anti—GM2 IgG/IgM	Negative	Negative
Anti—GM3 IgG	Negative	Negative
Anti GD1A	Negative	Negative
Anti GD1B	Negative	Negative
Anti GT1B	Negative	Negative
Anti GQ1B	Negative	Negative
Autoimmune meningitis serology		
NDMA Receptor Antibody	< 1:10	< 1:10
Anti—Ampiphysin	Negative	Negative
Anti—CV2	Negative	Negative
Anti—PNMA (Ma2/Ta)	Negative	Negative
Anti—Ri	Negative	Negative
Anti—Yo	Negative	Negative
Anti—Hu	Negative	Negative
Anti—Recoverin	Negative	Negative
Anti—SOX1	Negative	Negative
Anti—Titin	Negative	Negative
Systemic autoimmune disorder and vasculitis panel		
Serum C3 Complement	1.36 g/dL	0.9–1.8 g/dL
Serum C4 Complement	**0.49 g/dL**	**0.1–0.4 g/dL**
Rheumatoid Factor	3.02 IU/mL	< 14 IU/mL
IgA	3.37 g/L	0.7–4 g/L
IgE total	16 IU/mL	< 100 IU/mL
IgG	8.05 g/L	7–16 g/L
IgM	0.88 g/L	0.4–2.3 g/L
Anti‐RNP/Sm	1.8 U/mL	< 20 U/mL
Anti—Ro (SS‐A)	1.6 U/mL	< 20 U/mL
Anti—ds DNA	Negative	Negative
Anti—cardiolipin	3.3 U/mL	< 12 U/mL
Anti—LKM	Negative	Negative
p‐ANCA	1 U/mL	< 20 U/mL
c‐ANCA	11 U/mL	< 20 U/mL
Anti‐smooth muscle	Negative	Negative
Anti‐Sclero‐70	4 U/mL	< 20 U/mL
Anti‐beta2 glycoprotein 1 IgG	3.8 U/mL	< 16 U/mL
Anti‐mitochondrial antibodies	Negative	Negative
Circulating Immune Complexes	2.7 μmol/mL	< 16 μmol/mL

Further diagnostic evaluation included a cerebrospinal MRI with contrast, laboratory tests for immune‐mediated encephalopathies, and screening for systemic vasculitis. These investigations yielded negative results for N‐methyl‐d‐aspartate (NMDA) receptor antibodies, neuronal IgG antibodies, antiganglioside antibodies, and systemic vasculitis [4]. *A lumbar puncture performed in the first 3 days after the cardiac arrest (after a 24‐h discontinuation of therapeutic anticoagulation)* revealed albuminocytologic dissociation, with a cerebrospinal fluid (CSF) protein level of 63.2 mg/dL. While suggestive of an acute peripheral neuropathy, this finding is not uncommon in critically ill patients and did not confirm a definitive diagnosis. Viral and bacterial pathogens were not detected in the CSF or at other potential sites of infection (Table 2).

**TABLE 2:** CSF studies done over the course of the patient's stay in the ICU.

Parameter	Patient value	Reference range
Glucose	**139.73 mg/dL**	**40–70 mg/dL**
Total protein	**59.86 mg/dL**	**15–45 mg/dL**
WBC	1 elements/mm^3	< 3 elements/mm^3
CSF culture	Negative	Negative
NMDA Receptor Antibody	< 1:1	< 1:1
*CSF multiplex PCR*		
*E. coli* K1	Negative	Negative
*H. influenzae*	Negative	Negative
Listeria moncytogenes	Negative	Negative
*Neisseria meningitidis*	Negative	Negative
*S. agalactiae*	Negative	Negative
*S. pneumoniae*	Negative	Negative
CMV		
Enterovirus	Negative	Negative
Herpesvirus 1,2,6	Negative	Negative
Parechovirus	Negative	Negative
Varicella zoster	Negative	Negative
*Cryptococcus neoformans*	Negative	Negative

Electromyography (EMG), performed during the third week of ICU care, provided the crucial evidence for a diagnosis of GBS. The study demonstrated hallmark findings, including the sural‐sparing sensory phenomenon, an abnormal sensory nerve action potential (SNAP) of the median nerve, and severe, acute, symmetric motor axonal polyneuropathy affecting both proximal and distal segments. Based on these results, the neurology team confirmed the diagnosis of GBS. Due to the hyperacute onset, the team anticipated a worse prognosis and a prolonged recovery, which was communicated to the patient's family.

The diagnostic process was significantly hindered by logistical and resource‐related challenges. The patient's obesity necessitated specialized assistance for transport to the radiology department, causing delays in imaging studies. Furthermore, the absence of the hospital's only neurology technician skilled in EEG and EMG for most of the diagnostic period impeded timely evaluation, further complicating the differential diagnosis.

### 2.3 Therapeutic Intervention

Following confirmation of the diagnosis, the patient was started on a 3‐day course of intravenous immunoglobulin (IVIG) therapy at the standard dose of 0.4 g/kg/day. *The limited duration of treatment was dictated by financial constraints, which also demanded an objectified diagnosis of the disease for the neurologist in charge of the case to be able to prescribe and justify the costs of the treatment, considering that the cytolabuminologic dissociation observed is non‐specific and the antiganglioside antibodies were negative, even if the high possibility of GBS was taken into account since the beginning of the case*. Plasma exchange was not pursued due to the patient's persistent septic condition, which developed during the second and third weeks of ICU admission because of a malfunctioning bypass graft and subsequent peripheral ischemic complications.

Gradual motor recovery was noted beginning on the third day after completing the IVIG course. Although the initial progression of muscular weakness which was characterized by its peripheral‐to‐central pattern was not identified early, the typical reverse pattern of motor recovery, starting with major joints such as the shoulder girdle and hip joints, was observed. Unfortunately, recovery of motor function in the distal extremities remained limited at the time of discharge, reflecting the severity of the disease and the delayed intervention.

### 2.4 Follow‐Up and Outcomes

The patient required an additional 4 weeks of intensive care due to complications related to surgery. Ultimately, the ischemic lower limb necessitated above‐the‐knee amputation. Neurological recovery in the peripheral nervous system progressed slowly, supported by intensive physiotherapy. Importantly, there were no lasting central neurological deficits attributed to the initially suspected hypoxic encephalopathy. Mild cognitive impairments, including bradypsychia, bradylalia, and anterograde amnesia, were observed during the first few days after extubation but resolved completely over time.

Follow‐up assessments were conducted at 2 months post‐surgery, at the time of transfer to the ward, and again at 3 months following the initial hospitalization, when the patient was transferred to a rehabilitation unit. Moderate sensory and motor impairments persisted, consistent with the anticipated challenges of recovery given the severity of the disease and delayed diagnosis. These deficits continued to impact the patient's functional improvement, highlighting the complex nature of the rehabilitation process.

## 3 Discussion

Although GBS is classically characterized by a spectrum of neuropathic pathologies with progressive weakness and reduced or absent reflexes, the disease can rapidly progress to respiratory failure in approximately 20%–30% of cases [5]. In retrospect, the patient's cardiopulmonary arrest during the first days of ICU admission was most likely attributable to a hyperacute onset of GBS, with the nadir reached within less than 72 h. The surgery itself was the only identifiable precipitating event. *To be retained is also the extensive length of the surgery due to intra‐operative complications—graft thrombosis—and the subsequent inflammatory response, noticeable from the first 24 h*. It is plausible that the use of epidural analgesia may have obscured early neuropathic symptoms in this patient. Additionally, the surgical procedure could have acted as a significant trigger for the development of delirium [6], for which the patient was predisposed.

The doses of continuous intravenous sedation used to manage refractory delirium, which did not respond to standard preventive measures per our local protocol (a regimen combining a short‐acting benzodiazepine and a benzamide antipsychotic, followed by low‐dose intravenous propofol), were carefully titrated based on clinical response. This makes respiratory arrest from sedative overdose highly improbable. A systematic approach was undertaken to differentiate the potential causes of the patient's profound coma with flaccid quadriplegia, which became apparent shortly after resuscitation. The clinical presentation and subsequent diagnostic findings provided a rationale for excluding alternative etiologies and supported the diagnosis of GBS.

**FIGURE 2:**
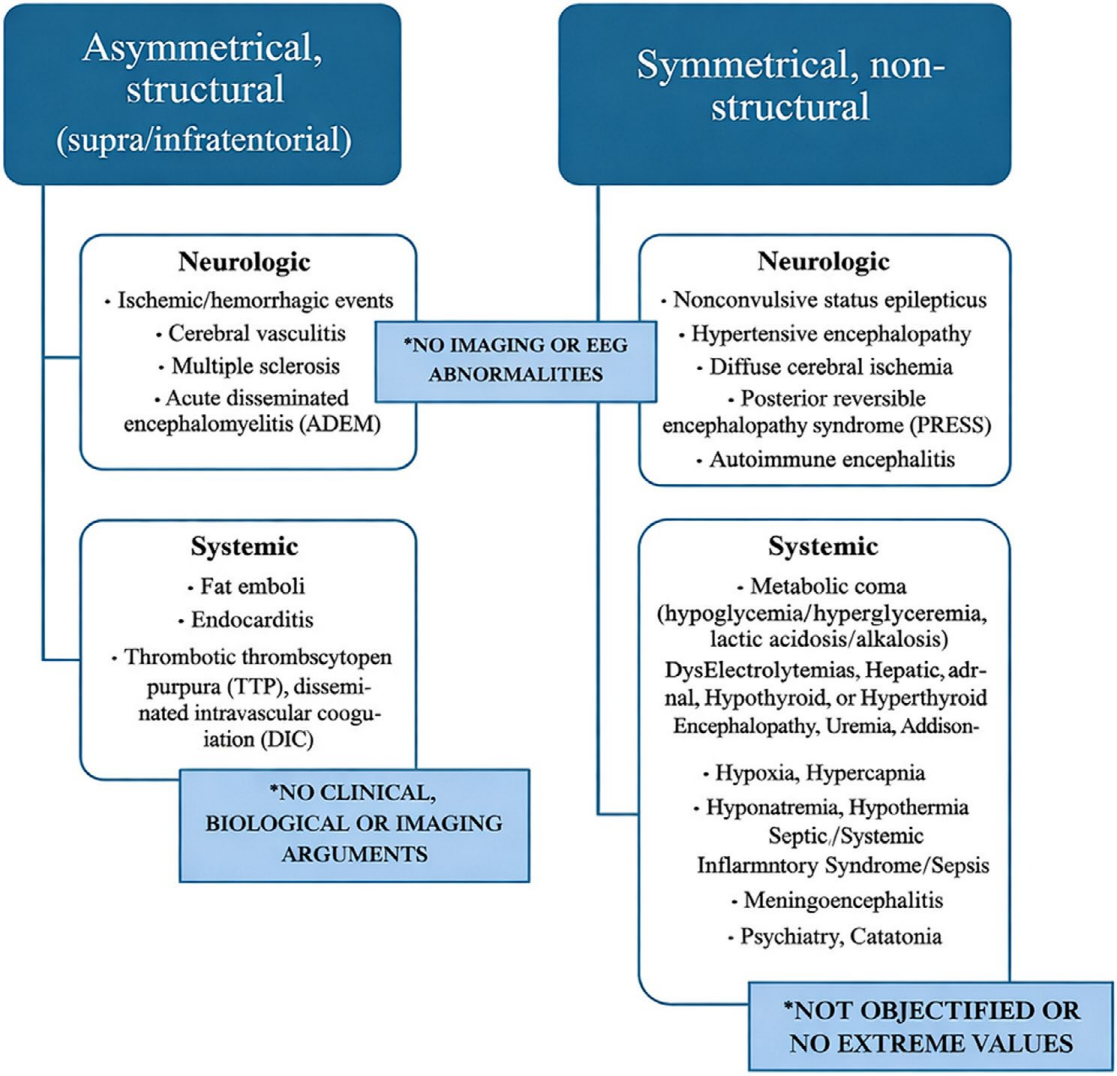
Differential diagnosis of coma and stupor in adults. Case specific etiology.

Coma in the ICU can be categorized into four main causes (Figure 2) [4]. Coma mimics include conditions like locked‐in syndrome, akinetic mutism, and catatonia, which resemble coma but do not impair consciousness. Systemic factors such as toxins, metabolic disturbances, infections, or environmental influences can alter neuronal oxygen delivery or consumption, leading to impaired awareness. Examples include cyanide poisoning, post‐anoxic encephalopathy, hypothermia, seizures, and neuroleptic malignant syndrome. Cerebral hemisphere damage typically involves bilateral, diffuse cortical injury, as seen in massive subarachnoid hemorrhage or diffuse axonal trauma. Severe unilateral lesions may also induce coma if they cause midline shift or brainstem compression. Finally, ARAS dysfunction arises from damage to infratentorial structures critical for consciousness, often caused by posterior circulation strokes, embolic events, trauma, or autoimmune disorders like Guillain‐Barré syndrome [7]. These categories provide a framework for diagnosing and managing coma in critically ill patients.

The differential diagnosis of coma‐like states involves ruling out mimics such as locked‐in syndrome, akinetic mutism, and akinetic catatonia. Locked‐in syndrome, caused by brainstem lesions like posterior circulation strokes, results in flaccid paralysis but preserves consciousness and vertical eye movement, with negative imaging helping to exclude it. Akinetic mutism, linked to prefrontal cortex lesions, features an awake state with preserved tone and reflexes, also ruled out by normal imaging. Akinetic catatonia, associated with psychiatric or medical conditions like schizophrenia and autoimmune disorders, is tested using the Lorazepam challenge, where a lack of response combined with EEG findings inconsistent with wakefulness confirms exclusion. These steps are essential for identifying the true cause of coma‐like presentations.

Toxic, metabolic, infectious, and environmental factors are essential considerations in coma differential diagnosis. Sepsis‐associated encephalopathy (SAE) is a common but poorly understood condition linked to systemic infection without direct CNS involvement, presenting with varying severity from mild cognitive deficits to coma [8]. Diagnosis relies on exclusion due to a lack of specific biomarkers, though in this case, the rapid onset of neurological symptoms before sepsis development excluded SAE. Toxic encephalopathy from drugs like antipsychotics or serotoninergic agents, which can cause neuroleptic malignant syndrome or serotonin syndrome, respectively, was ruled out as the anesthetic record did not indicate their use [9]. Similarly, hypothyroidism, known to induce coma through metabolic disruptions and altered medication metabolism, was excluded based on normal thyroid function tests and the absence of patterns consistent with euthyroid sick syndrome.

Damage to the cerebral hemispheres or the brainstem's ARAS‐associated structures was considered as potential causes of the patient's coma. Bilateral, diffuse cerebral damage from causes such as CVAs, cerebral edema, tumors, or trauma is a common etiology for coma, especially in patients with extensive cardiovascular histories. However, repeated imaging over 72 h, including contrast‐enhanced CT and MRI, showed no cerebral lesions, confidently excluding these pathologies. Brainstem involvement, specifically posterior circulation CVAs, was a key consideration due to its association with ARAS dysfunction. While the initial CT scan was negative, its limited sensitivity for posterior fossa lesions prompted a cerebrospinal MRI, which also revealed no macroscopic abnormalities [10]. This left microscopic, diffuse ARAS lesions, such as infectious or autoimmune etiologies, as the most plausible explanation. Subsequent blood and CSF cultures, performed after suspending antibiotics and correcting coagulation status, were negative, effectively ruling out systemic and neuroinfections.

The biochemical analysis of the CSF revealed a slight elevation in CSF protein (63.8 mg/dL, with a reference range of 15–60 mg/dL), which is a common finding in both general and ICU populations [11]. It also showed albumino‐cytologic dissociation, characterized by increased CSF total protein with a white cell count < 50 × 10^9/L. While this finding is sensitive for an autoimmune process, it lacks specificity [12]. Combined with the negative results from other investigations, these modest positive findings suggest an autoimmune etiology, possibly an autoimmune encephalitis or a neuropathy with atypical central involvement. Table 3 lists the various encephalitis‐associated antibodies and their diagnostic significance [13].

**TABLE 3:** Autoimmune encephalitides, their associated antibodies and the most commonly associated cancers.

Antibody	Syndrome	Associated cancer/illness
Antibodies against intracellular antigens		
Hu	Limbic encephalitis	Small‐cell lung carcinoma
Ma2	Limbic encephalitis	Testicular seminoma
GAD	Limbic encephalitis	Thymoma, small‐cell lung carcinoma
Antibodies against synaptic receptors		
NMDA	Anti‐NMDA encephalitis	Ovarian Teratoma, Herpetic encephalitis
AMPA	Limbic encephalitis	Thymoma, Small‐cell lung carcinoma
GABA‐B	Limbic encephalitis	Thymoma
Dopamine D2	Basal ganglia encephalitis	—
Antibodies against ion channels/other surface proteins		
GQ1b	Bickerstaff's brainstem encephalitis	—
MOG	ADEM	Viral infection usually under 40 years
CASPR2	Limbic encephalitis	Thymoma
LGI1	Limbic encephalitis	Thymoma

Existing diagnostic criteria for autoimmune encephalitis largely depend on antibody testing and the response to immunotherapy. However, antibody testing is not always readily available, and results may take weeks. Moreover, the absence of autoantibodies does not rule out an immune‐mediated disorder, and a positive result does not always confirm the diagnosis [13]. In our case, GBS variants are particularly relevant, given their diverse presentations. The most common GBS variant is Acute Inflammatory Demyelinating Polyneuropathy (AIDP), characterized by subacute onset and progressive, symmetric weakness, often following gastroenteritis. Acute Motor Axonal Neuropathy (AMAN) involves selective motor nerve impairment with sensory sparing and is more common in Asia. Acute Sensory and Motor Axonal Neuropathy (ASMAN) is the most severe axonal variant, leading to permanent sensory damage. The anti‐GQ1B syndromes include variants like Miller‐Fisher Syndrome and Bickerstaff Brainstem Encephalitis, linked to antibodies against gangliosides in cranial nerves, affecting brainstem function [14]. Hyperacute/Fulminant GBS is the rarest form, presenting with rapid progression to quadriplegia and respiratory paralysis, often without CSF abnormalities [15, 16].

The clinical suspicion of GBS is typically raised by a history of an acute respiratory infection or gastroenteritis, along with the classic signs of distal muscle weakness and areflexia. While these findings are common in AIDP, the most frequent GBS variant in Europe and the US, they are highly variable in other forms of the disease. Additionally, numerous cases of GBS have been reported without an identifiable infectious trigger [2]. Respiratory failure as an initial symptom makes GBS less likely, according to the European Academy of Neurology's guidelines, highlighting the need for a more refined diagnostic approach to recognize the disease's heterogeneous nature [17]. CSF analysis usually reveals albuminocytological dissociation, indicating disruption of the blood‐nerve and blood‐CSF barriers [12]. CSF serology can detect antigangliosidic antibodies, which help confirm the diagnosis and identify specific GBS variants, offering clues to prognosis. Table 4 offers an overview of the different antibodies involved in the variants of GBS [18]. However, these antibodies are found in only 60% of GBS cases [18].

**TABLE 4:** A classification of the variants of GBS according to clinical findings and the most common associated CSF antibodies.

Classification	Clinical features	Antibodies present
*AIDP*	Paresthesia, weakness	AntiGM1, antiGD1a
*AMAN*	Weakness, no paresthesia, less reversible	AntiGD1a, antiGM1
*ASMAN*	Paresthesia, weakness, less reversible	AntiGM1, AntiGM1b, AntiGD1a
*PCB*	Bulbar, cervical, and upper limb reversible weakness and paresthesia	AntiGQ1b, AntiGT1a
*MFS*	Ophthalmoplegia, ataxia, areflexia/hyporeflexia	AntiGQ1b, antiGT1a
*BBE*	Hypersomnolence, Ophtalmoplegia, Ataxia, no limb weakness	AntiGQ1b, antiGD1b

Abbreviations: AIDP, Acute Inflammatory Demyelinating Polyradiculoneuropathy; AMAN, Acute Motor Axonal Neuropathy; ASMAN, Acute Sensory and Motor Axonal Neuropathy; BBE, Bickerstaff Brainstem Encephalitis; MFS, Miller Fisher Syndrome; PCB, Pharyngeal‐Cervical‐Brachial variant.

The most representative Nerve Conduction Studies (NCS) findings in GBS include motor and/or sensory nerve conduction slowing, block, prolonged distal motor latency (DML), and absent F‐waves. However, these findings may be normal in the early stages of the disease, making serial NCS tests crucial for an accurate diagnosis. While traditional methods emphasized multiple NCS tests, a 2020 study by Rajabally and colleagues showed that a single early NCS can reliably diagnose GBS [19]. A key diagnostic finding in this patient was the Sural Sparing Sensory Phenomenon, where normal sensory neural action potentials (SNAPs) are detected in the sural nerve, while upper limb nerve SNAPs show lower amplitude. This phenomenon suggests a lower degree of blood‐nerve barrier disruption in the sural nerve, helping differentiate GBS from critical illness polyneuropathy, which shares similar electrodiagnostic features. The patient's pattern of severe proximal and distal motor axonopathy with sensory impairment, along with sural sparing, indicated a fulminant form of Acute Sensory and Motor Axonal Neuropathy (ASMAN) with central involvement. Given the underlying axonal disruption, full neurological recovery is unlikely.

### 3.1 Brighton Scale Detailed

The European Academy of Neurology guidelines for treating GBS focus on four key interventions: ICU admission, respiratory care, immunotherapy, and rehabilitation [20]. Patients at risk of respiratory decline should be admitted to the ICU, with the Modified Erasmus GBS Respiratory Insufficiency Score (mEGRIS) helping assess the need for respiratory support. Immunotherapy, using IVIG or plasma exchange, is the primary treatment to halt disease progression, with corticosteroids offering no benefit. IVIG is recommended at 0.4 g/kg/day for 5 days for patients unable to walk, with no repeat courses suggested. Given concerns about systemic infection, the patient was treated with IVIG, although only a 3‐day course was administered due to limited availability at our institution. For pain management, neuropathic pain agents such as carbamazepine and gabapentinoids were recommended, starting in the acute phase and continuing through recovery. Rehabilitation, which is advised for all patients from the acute phase onwards, was not feasible in this case due to a lack of appropriate neurorecovery services within the healthcare system, and no private institutions were available for this purpose.

## 4 Conclusion

The hyperacute presentation of GBS is a rarely documented and fulminant medical emergency that often presents with atypical symptoms, requiring a broad differential diagnosis. In this case, the initial encephalopathic symptoms of hyperacute GBS posed a significant challenge due to its rarity, prompting our team to systematically rule out other potential causes of CNS dysfunction. This case report highlights the complexities of diagnosis, influenced by the patient's comorbidities and hospital infrastructure, as well as the difficulties encountered in providing optimal treatment.

## Author Contributions

**Miron Tiganas:** conceptualization, data curation, formal analysis, investigation, resources, writing – original draft. **Abdulrahman Ismaiel:** data curation, formal analysis, supervision, visualization, writing – original draft, writing – review and editing. **Dan Sebastian Dirzu:** supervision, writing – review and editing. **Iris Aszalos:** conceptualization, data curation, investigation, project administration, resources, supervision, writing – review and editing.

## Funding

The authors have nothing to report.

## Consent

This case report is published with the written consent of the patient.

## Conflicts of Interest

The authors declare no conflicts of interest.

## Data Availability

Information can be provided on reasonable request by contacting the corresponding author.
